# Transcriptomic profiling of long non-coding RNAs in hepatitis B virus-related hepatocellular carcinoma

**DOI:** 10.18632/oncotarget.18897

**Published:** 2017-06-30

**Authors:** Hongxia Fan, Qiaoge Zhang, Xiaopei Zhao, Ping Lv, Min Liu, Hua Tang

**Affiliations:** ^1^ Tianjin Life Science Research Center and Department of Pathogen Biology, School of Basic Medical Sciences, Tianjin Medical University, Tianjin, China

**Keywords:** HBV, long non-coding RNAs, hepatocellular carcinoma, RNA sequencing, n346077

## Abstract

Long non-coding RNAs (lncRNAs) have been reported to be involved in the development and progression of hepatocellular carcinoma (HCC). However, few studies have focus on the dyregulation and the role of lncRNAs in HBV-related HCC. We performed a comprehensive analysis of lncRNAs expression profile in HBV-related HCC tissues samples using deep sequencing. We revealed that a total of 1242 lncRNA transcripts (983 up-regulated and 259 down-regulated) and 1841 mRNA transcripts were significantly differentially expressed in HBV-related HCC patients. Pathway and gene ontology analysis showed that they are involved in the biological process related to HCC development by cis-regulation of co-expressed protein-coding genes. 10 candidate lncRNAs were selected and validated with quantitative real-time PCR analysis. Furthermore, we found that one of most down-regulated lncRNAs, n346077, could suppress HCC cells invasion and migration *in vitro*. Our findings provide an overview of aberrantly expressed lncRNAs in HBV-related HCC and will be useful for further functional studies of lncRNAs in HBV-related pathogenesis.

## INTRODUCTION

HCC is one of the most common cancers and is the second leading cause of cancer-related mortality worldwide [1]. The risk factors accounting for HCC development include chronic HBV infection [2], hepatitis C virus infection [3], excessive consumption of alcohol [4], and aflatoxin exposure [5]. Among them, HBV is a major etiological agent of HCCs. HBV contributes to HCC formation and development through direct and indirect pathways. The prolonged expression of the viral regulatory protein HBx and/or altered versions of the preS/S envelope proteins deregulate cell transcription, alter cells proliferation control and sensitize liver cells to carcinogenic factors. The integration of HBV DNA into the host genome induces genomic instability and eventually direct insertional mutagenesis of diverse cancer-related genes [6, 7]. But the detailed mechanism underlying HBV induced-HCC is still unclear.

The recent developments in deep sequencing technologies have led to the detection of non-coding RNAs which comprised the majority of the transcriptome. Non-coding RNAs can be generally divided into two classes according to their size: small non-coding RNAs (e.g., microRNAs) and long non-coding RNAs (e.g., lncRNAs) [8, 9]. lncRNAs are a group of non-coding RNAs longer than 200 nucleotides in length with little or no protein-coding capacity. According to the proximity to nearby coding genes, lncRNAs could be categorized to 5 classes: sense, antisense, intergenic, bidirectional and intronic [9]. lncRNAs can promote/suppress gene expression at epigenetic, transcriptional, and post-translational levels [10], and are involved in various biological processes across every branch of life including cell growth, differentiation, proliferation, survival, migration and so on [10]. Notably, emerging data have showed that the mutation and dysregulation of lncRNAs can result in aberrant expression of gene products that contribute to the progress of various human diseases, especially cancer [10, 11]. Several lncRNAs have been found to contribute to the pathologic process of HCC by multiple mechanisms [12, 13]. Among them, the lncRNAs highly up-regulated in liver cancer (HULC), high expression in HCC (HEIH), HBx-interspersed nuclear element 1 (HBx-LINE1), H19, HOTAIR, Unigene56159, down-regulated expression by HBx (DREH) and microvascular invasion in HCC (MVIH) are strongly implicated in the development of HBV-related HCC [14–21]. For example, the lncRNA HEIH was highly expressed in the patients with HBV-related HCC. It promoted cell proliferation through repressing the targets genes of enhancer of zeste homolog 2 (EZH2) [15]. HBx-LINE1 was a viral-human chimeric transcript identified from transcriptome sequencing of HBV-positive HCC cell lines. It functions like a long non-coding RNA to promote HCC through activating Wnt/β-catenin signaling pathway [16]. In our recent study, we found that Unigene56159, which is highly expressed in HBV-related HCC tissues, promoted HCC cells migration and invasion by acting as a competing endogenous RNA (ceRNA) for miR-140-5p to de-repress the expression of the target gene Slug [19]. However, few studies have focus on the lncRNA expression profile in HBV-related HCC and the exact role of lncRNAs in HBV-related HCC has not yet been clearly clarified.

In the present study, to identify the lncRNAs that are participated in the progress of HBV-related HCC, we investigated the lncRNA expression profile in HBV-positive HCC (HBV (+) HCC) and HBV-negative (HBV (–) HCC) using high-throughput RNA sequencing (RNA-Seq) and annotated their functions with gene ontology (GO) analysis and Kyoto Encyclopedia of Genes and Genomes (KEGG) pathway analysis of their cis- and trans-regulated protein-coding genes. Furthermore, the function of n346077, a lncRNA was markedly down-regulated in HBV (+) HCC, was characterized *in vitro*. To the best of our knowledge, this is the first report of a comprehensive identification of lncRNAs in HBV-related HCC using RNA-seq analysis, which will facilitate our understanding of the roles of lncRNAs in the development of HBV-related HCC.

## RESULTS

### RNA-seq analysis and identification of mRNAs and lncRNAs in HBV-related HCC

**Figure 1:**
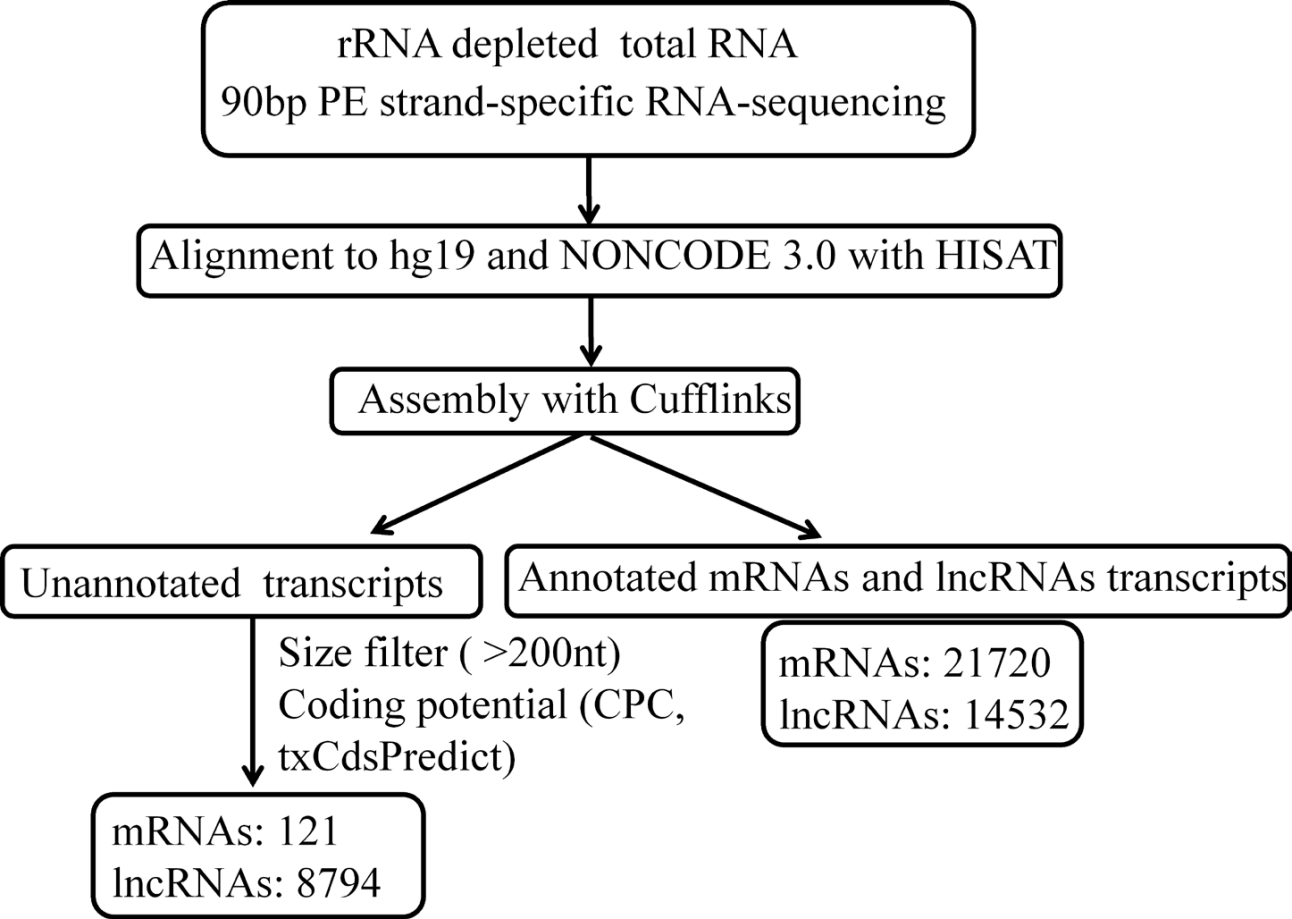
The pipeline for systematic identification of lncRNAs and mRNAs in HBV-related HCC CPC, Coding Potential Calculator.

To comprehensively identify lncRNAs and mRNAs associated with HBV-related HCC, we performed the whole transcriptome strand-specific RNA-Seq on rRNA-depleted RNAs from 3 HBV (+) HCC and 3 HBV (–) HCC tissue samples. A total of 199.4 million clean reads were obtained from sequencing. More than 179.7 million read pairs (90.1%) were aligned to the human genome (hg19). The mapped reads were used for assembling putative lncRNAs with cufflink program and the assembled transcripts were annotated. For mRNAs, RefSeq database (Build 38) was chosen as the annotation reference. For lncRNAs, NONCODE v3 database was chosen as the annotation reference. Through the transcripts identification pipeline (Figure 1), we obtained 21841mRNA isoforms derived from 16662 gene loci and 23326 expressed lncRNA isoforms derived from 23289 gene loci. Furthermore, 8794 (37.60%) of all 23326 lncRNAs were not identified in the NONCODE v3 lncRNA annotation.

**Figure 2:**
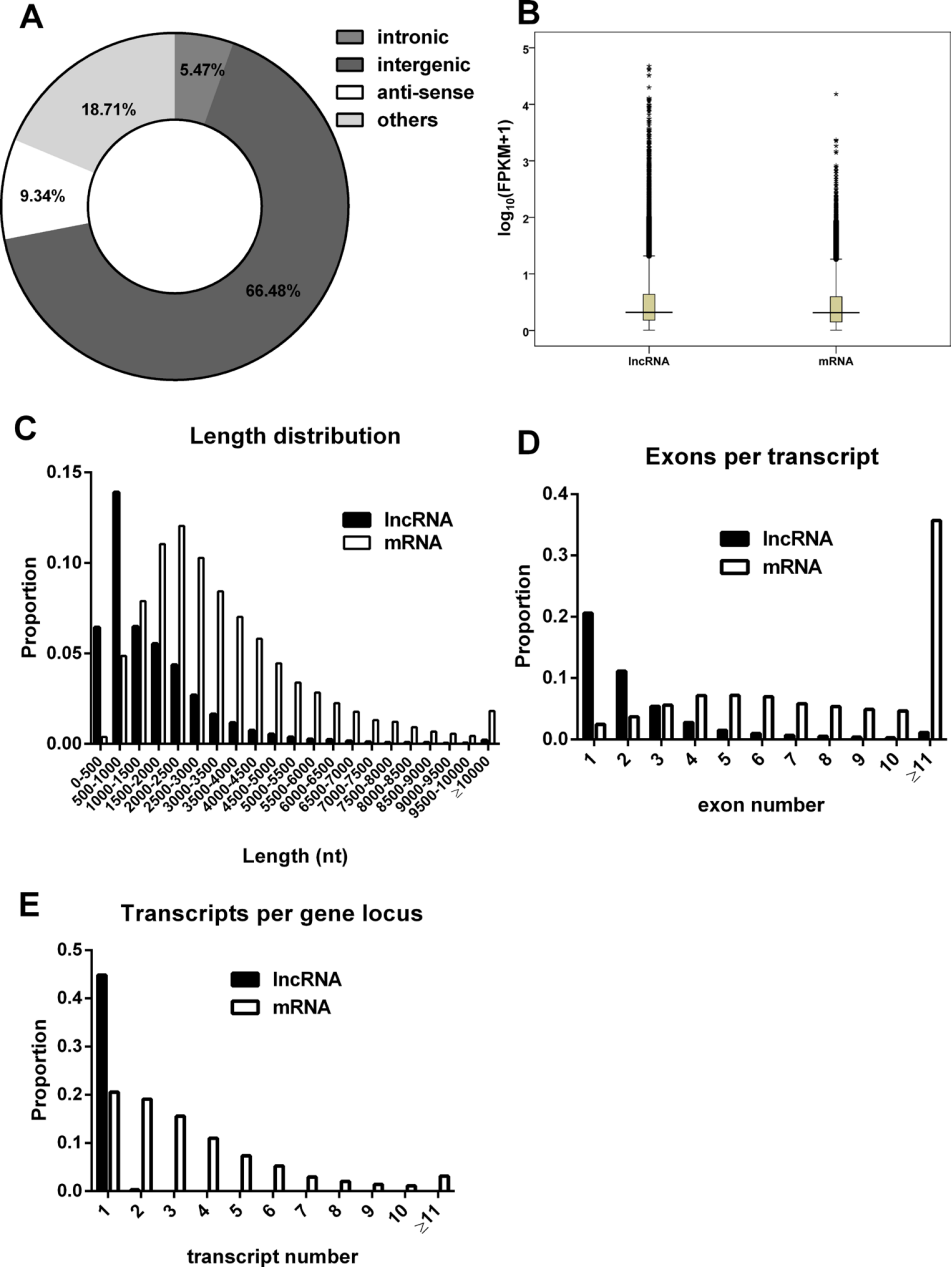
Characteristics of the lncRNAs (**A**) Classification of lncRNAs. (**B**) The expression levels indicated by log10 (FPKM + 1) for mRNAs and lncRNAs transcripts. (**C**) The length distributions of lncRNA and mRNA transcripts. (**D**) The number of exons per transcript for lncRNA and mRNA. (**E**) The number of isoforms per gene locus for lncRNAs and mRNAs.

We characterized the basic features of the lncRNAs and compared them with protein-coding genes where appropriate. Based on their relative genomic locations to neighboring coding genes, the lncRNAs include 15506 long intergenic lncRNAs, 1277 intronic lncRNAs, 2179 antisense lncRNAs and 4364 others (Figure 2A). Our data showed that the average lncRNAs expression levels were slightly higher than protein-coding gene expression levels (Figure 2B). As shown in Figure 2C, lncRNA transcripts are shorter in length than protein coding transcripts. Moreover, the exons number of lncRNAs was also less than that of mRNAs (Figure 2D). Both lncRNAs and mRNAs were alternatively spliced. On average, there were 1.01 isoforms per lncRNA gene loci and 3.49 isoforms per mRNA gene loci (Figure 2E).

### Differentially expressed mRNAs and lncRNAs in HBV-related HCC

**Figure 3:**
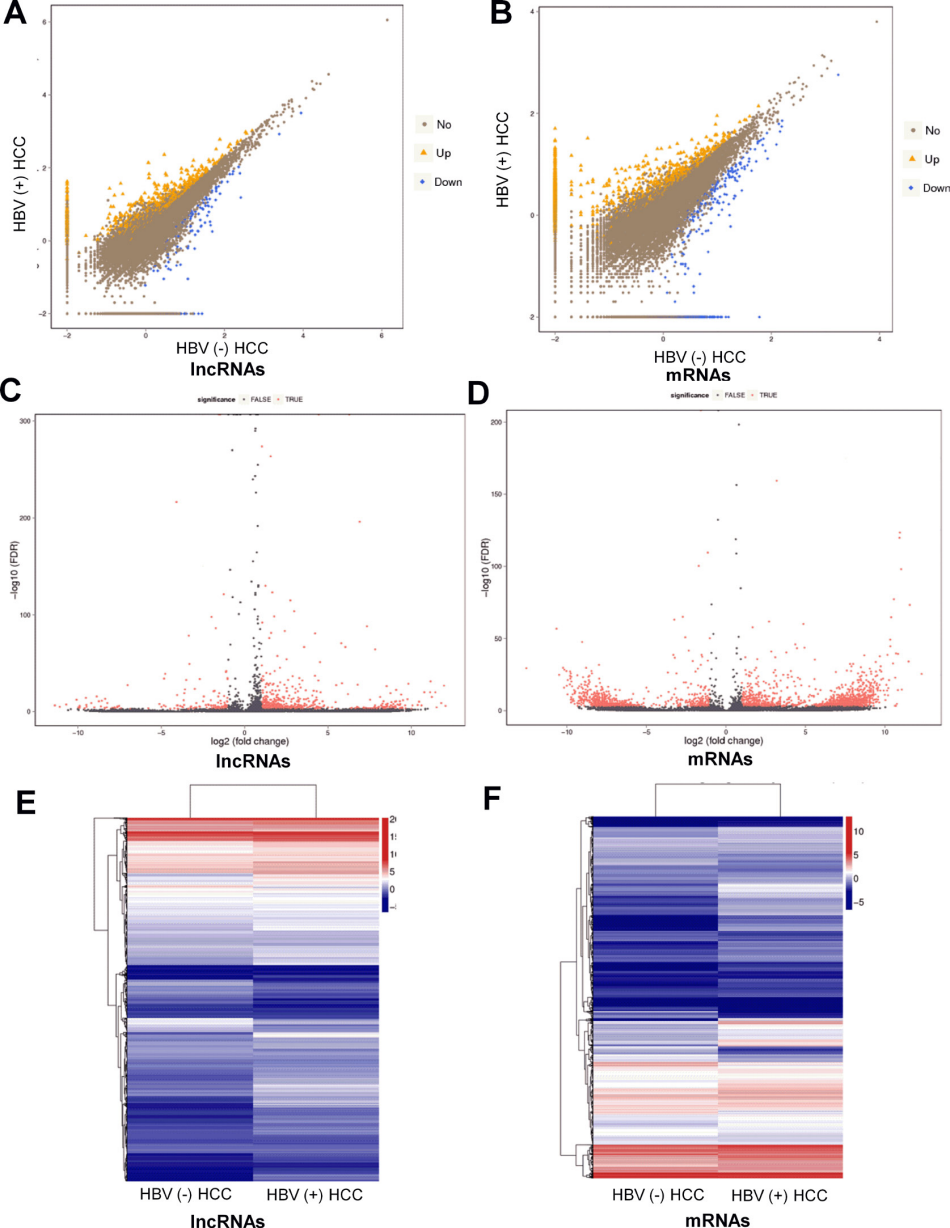
Differentially expressed lncRNAs and mRNAs between HBV (+) HCC and HBV (–) HCC tissue samples (**A**) and (**B**) Scatter plots of lncRNA and mRNA transcripts expression. The values of X and Y axes are the normalized signal values of groups (log2 scaled). The symbol triangle and diamond indicated more than 2 fold change between two groups. (**C**) and (**D**) Volcano plot of differentially expressed lncRNAs and mRNAs transcripts. The red points represent the differentially expressed genes with statistical significance. (**E**) and (**F**) Hierarchical clustering of the expression profiles of differentially expressed lncRNAs and mRNAs transcripts. Red indicates high relative expression and blue low relative expression.

Volcano plot, scatter plots and hierarchical clustering analysis were performed to assess gene expression variation between HBV (+) HCC tissues and HBV (–) HCC tissues (Figure 3). lncRNAs and mRNAs with at least 2 fold expression change were identified as significantly differently expressed (*p <* 0.05, FDR ≤ 0.001). Data analysis revealed 1242 lncRNA transcripts and 1841 mRNA transcripts were differentially expressed in HBV (+) HCC tissues compared to HBV (–) HCC tissues. Of the 1242 differentially expressed lncRNA transcripts, 983 were up-regulated and 259 were down-regulated. Of the 1841 differentially expressed mRNA transcripts, 1191 were up-regulated and 650 were down-regulated.

### Validation of dysregulated lncRNA expression in HBV (+) HCC patients and HBV (+) cell line

**Figure 4:**
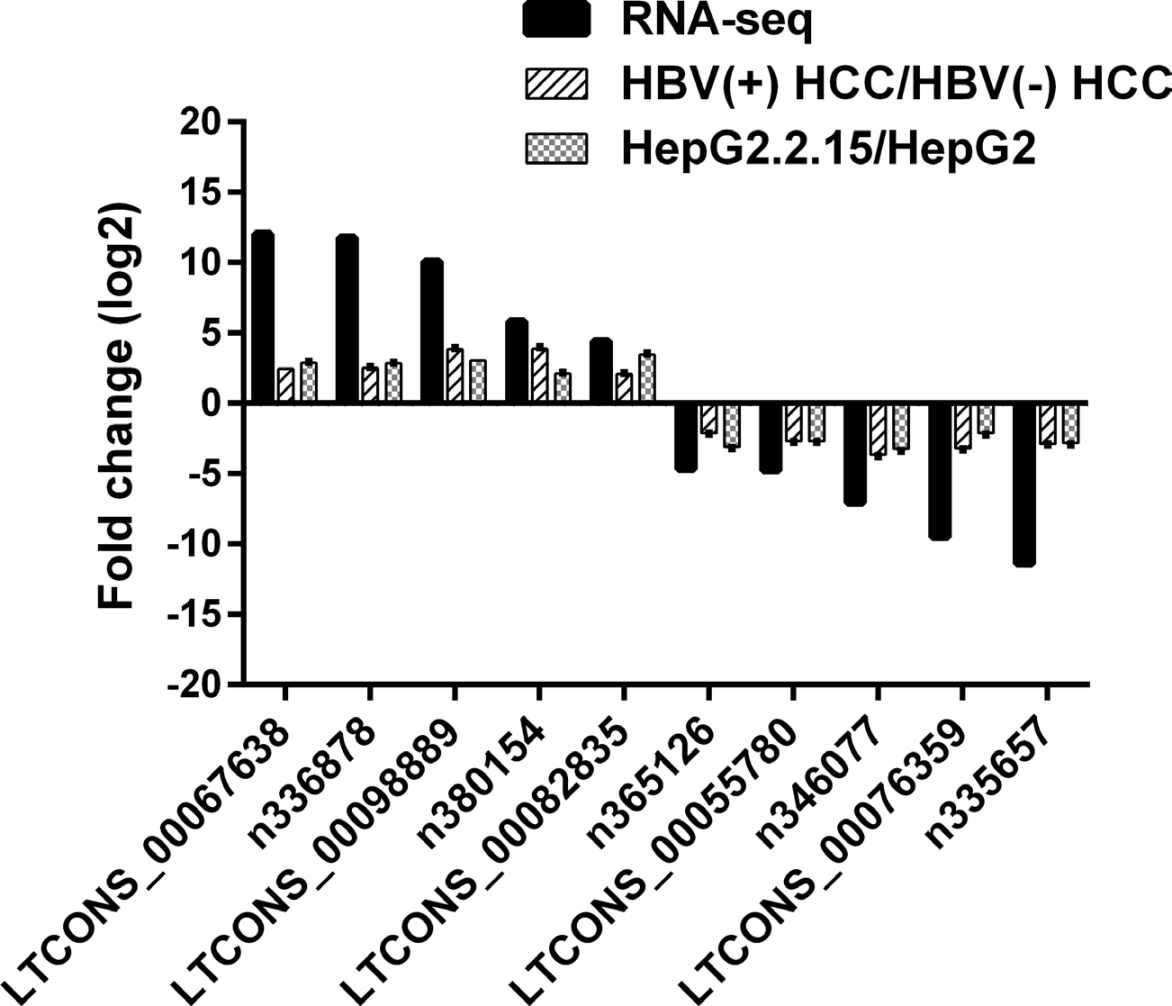
Validation for the expression of 10 randomly selected lncRNAs using RT-qPCR RT-qPCR analysis of RNA extracted from 10 HBV (+) HCC patients and 3 HBV (–) HCC patients or HepG2.2.15 and HepG2 cells. β-Actin was used as an internal control. Each sample was analyzed in triplicate. The heights of column represent mean fold changes (log2 transformed) compared with control groups.

To validate our RNA-seq data, we randomly selected 5 up-regulated and 5 down-regulated lncRNAs and analyzed their expression levels in another independent cohort of 10 HBV (+) HCC patients and in HepG2.2.15 cells containing dimers of HBV genomic sequence that could constitutively produce HBV particles with quantitative real-time PCR (RT-qPCR). As shown in Figure 4, compared to HBV (–) HCC, the selected lncRNAs displayed the same expression trend with the RNA-Seq data. Similar result was observed in HepG2.2.15 cells. These data verified the reliability of the RNA-Seq results.

### Functional annotation of differentially expressed mRNAs

**Figure 5:**
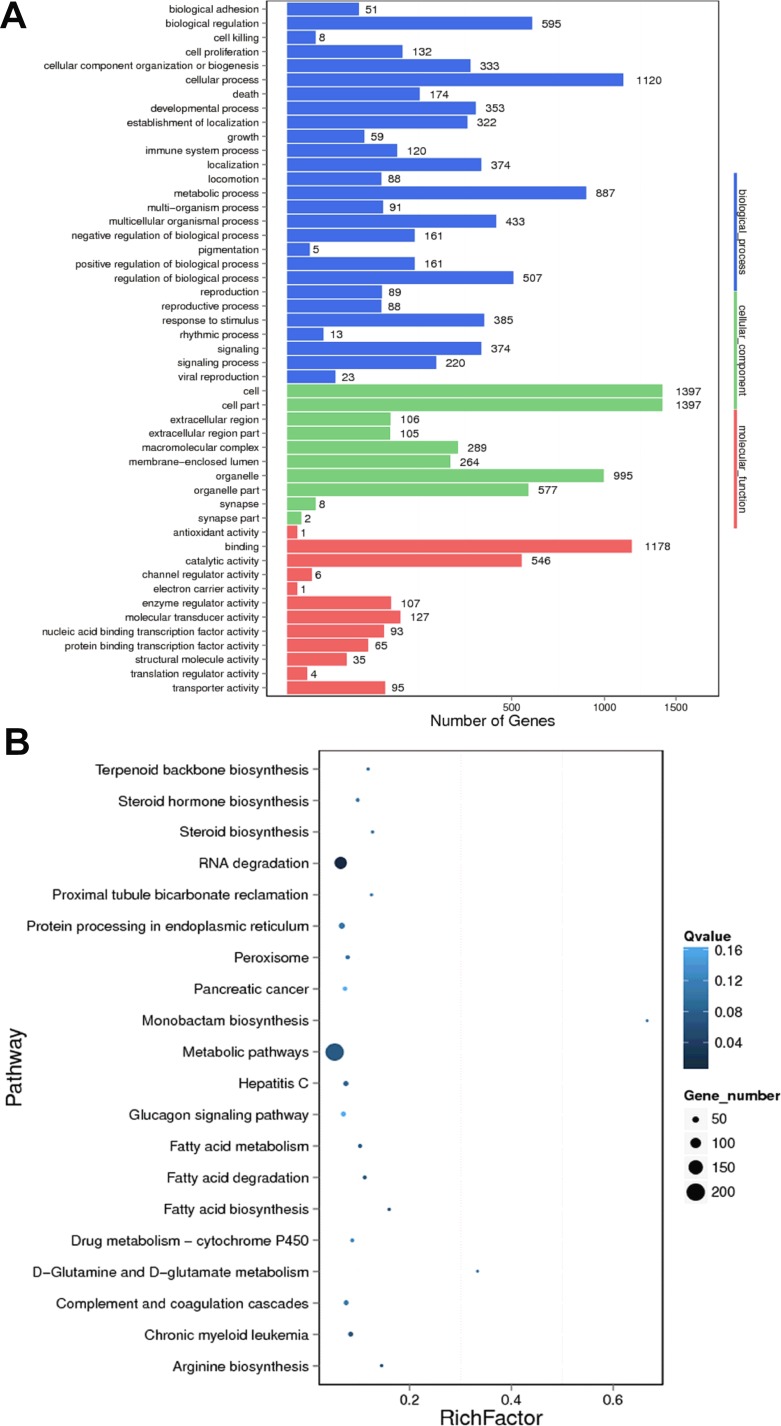
GO and KEGG pathway analysis of differentially expressed mRNAs between HBV (+) HCC and HBV (–) HCC tissue samples (**A**) The Go terms covering domains of biological processes, cellular components and molecular functions enriched among up- and down-regulated mRNAs. (**B**) Top 20 enriched pathways among up- and down-regulated mRNAs.

GO analysis for the differentially expressed mRNAs was performed to identify their function. The top 10 enriched GO items were listed in Figure 5A, including cell, cell part, binding, cellular process, organelle, metabolic process, biological regulation, organelle part, catalytic activity, regulation of biological process. The KEGG pathway analysis revealed that the most enriched pathways included RNA degradation, fatty acid degradation, chronic myeloid leukemia and metabolic pathway (Figure 5B).

### Prediction of lncRNA function

**Figure 6:**
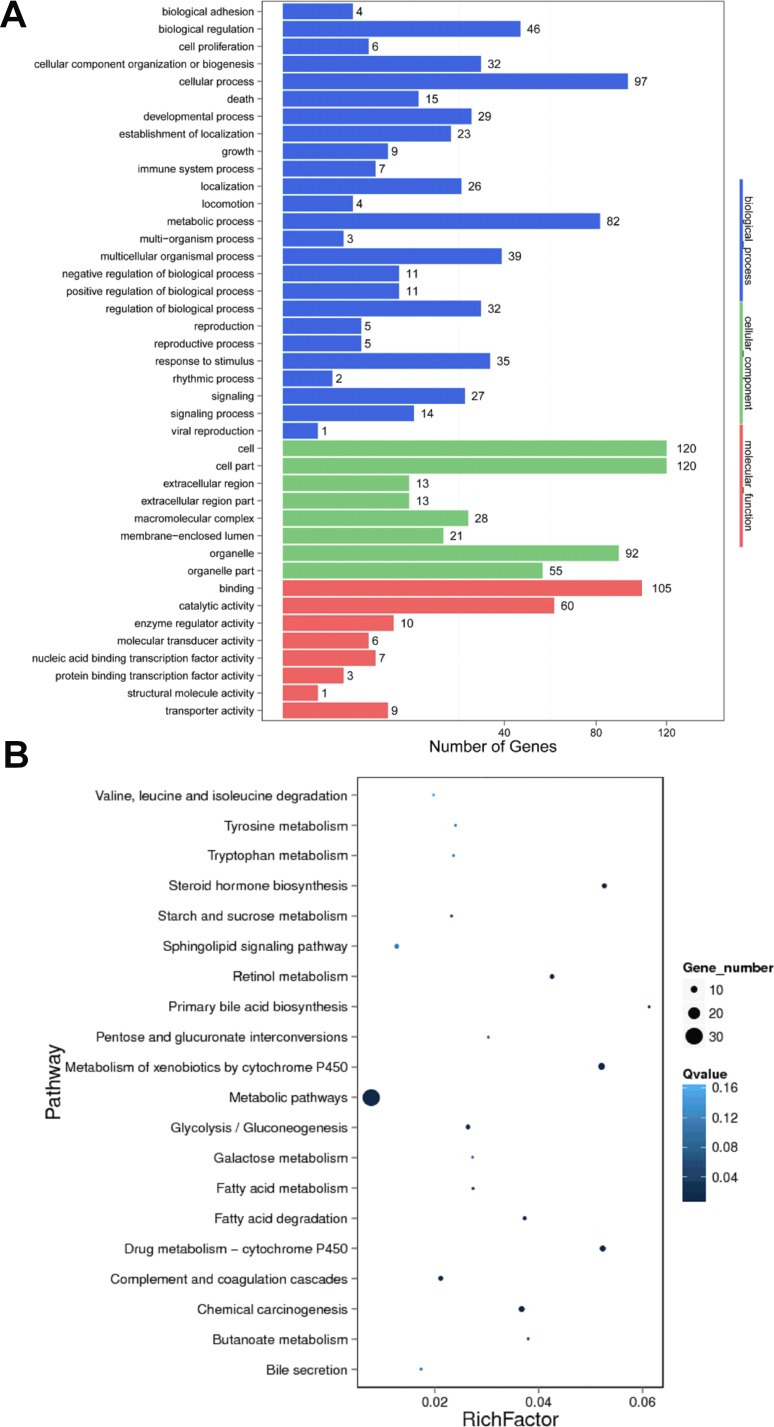
GO and KEGG pathway analysis of target genes of differentially expressed lncRNAs between HBV (+) HCC and HBV (–) HCC tissue samples (**A**) GO analysis of predicted cis-regulated target protein-coding genes of differentially expressed lncRNAs. (**B**) KEGG pathway analysis of predicted cis-regulated target protein-coding genes of differentially expressed lncRNAs.

To evaluate the function of differentially expressed lncRNAs, we predicted their cis-regulated target genes by search for protein-coding genes 10 and 100 kb upstream and downstream of the lncRNAs, respectively. And then performed GO and KEGG analysis on these cis-regulated target genes. GO analysis demonstrated that the significantly over-represented terms are similar to that for differentially expressed mRNAs (Figure 6A). KEGG pathway analysis showed that these cis-regulated target genes were enriched in metabolism of xenobiotics by cytochrome P450, drug metabolism - cytochrome P450, chemical carcinogenesis, steroid hormone biosynthesis, retinol metabolism, metabolic pathways, fatty acid degradation, glycolysis/gluconeogenesis, complement and coagulation cascades and primary bile acid biosynthesis (Figure 6B). Surprisingly, based on our prediction criterion, there was no trans-regulated target gene for the differentially expressed lncRNAs.

### lncRNA n346077 suppresses HCC cells migration and invasion

**Figure 7:**
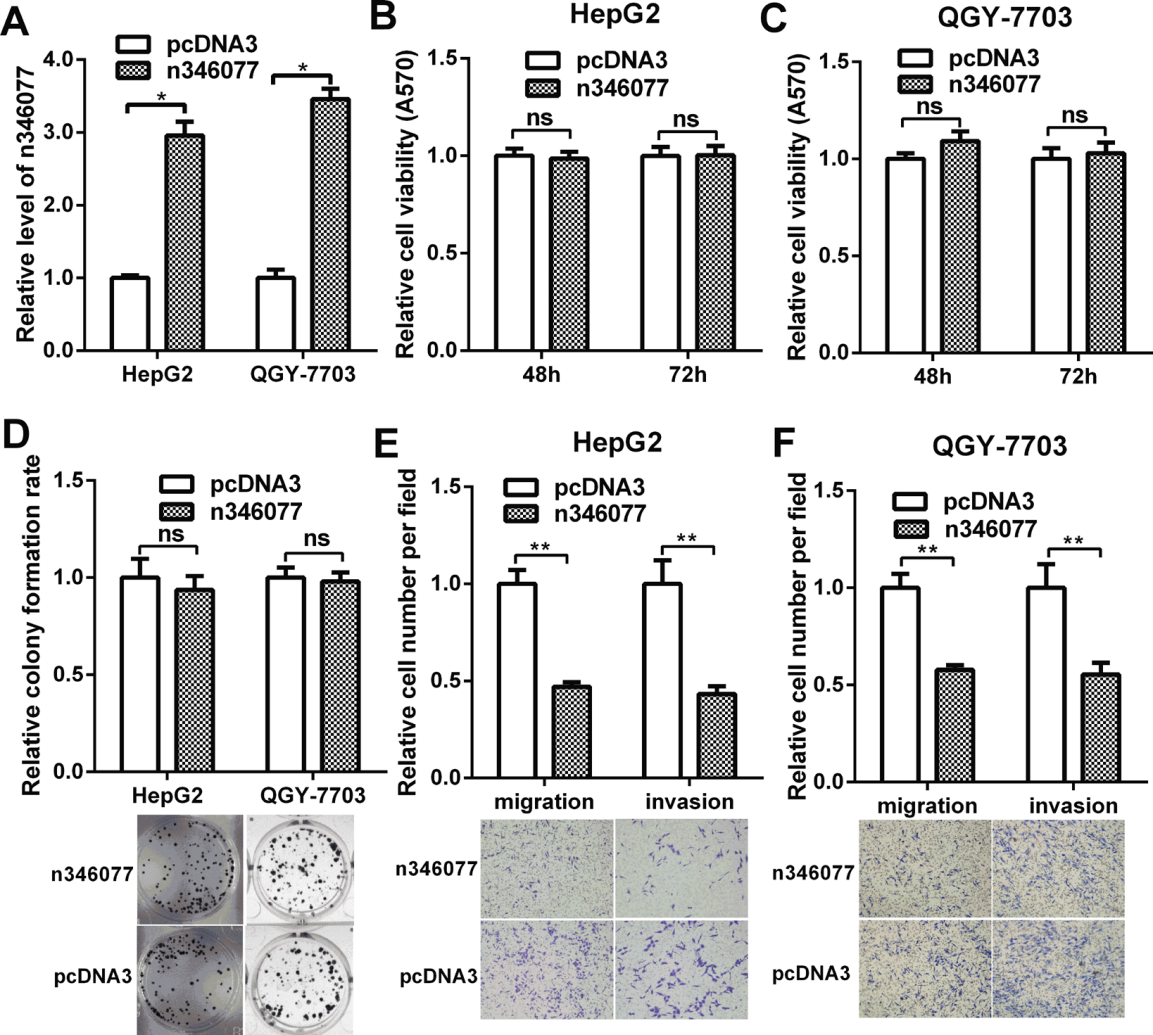
The effect of n346077 overexpression on malignant behavior of HCC cells *in vitro* (**A**) The expression level of n346077 was analyzed by RT-qPCR in HepG2 and QGY-7703 cells after transfection with n346077 overexpression plasmid. (**B**) and (**C**) Cell viability was measured in both HepG2 and QGY-7703 cells at 48 h, 72 h after transfection with n346077 overexpression plasmid by MTT assay. (**D**) Colony formation assay was performed in both HepG2 and QGY-7703 cells after transfection with n346077. Representative images are shown. (**E**) and (**F**) The migration and invasion abilities were determined both in HepG2 and QGY-7703 cells after transfection with n346077 by Transwell migration and invasion assays. Cells in three random fields of view at 100× magnification were counted and expressed as the average number of cells per field. Representative images are shown. *P < 0.05; **P < 0.01.

n346077, which encodes a 2609bp transcript and located in the opposite strand of mitochondrial ribosomal protein L23 (MRPL23) gene on chromosome 11, is one of the down-regulated lncRNAs, to probe the potential role of it in HCC cells, we first performed MTT, colony formation, cell migration, and invasiveness assays in HepG2 and QGY-7703 cells with n346077 overexpression. The efficiency of the n346077 overexpression plasmid was confirmed in HepG2 and QGY-7703 cells (Figure 7A). As shown in Figure 7B–7D, there was no significant difference in the viability and colon formation ability of HepG2 and QGY-7703 cells when n346077 was overexpressed compared to control. But the results of transwell assay with or without Matrigel showed that the cell migration and invasion abilities of both HepG2 and QGY-7703 cells were significantly suppressed after transfection with n346077 (Figure 7E and Figure 7F). These data indicated that n346077 suppresses cell invasion and migration of HCC cells.

**Figure 8:**
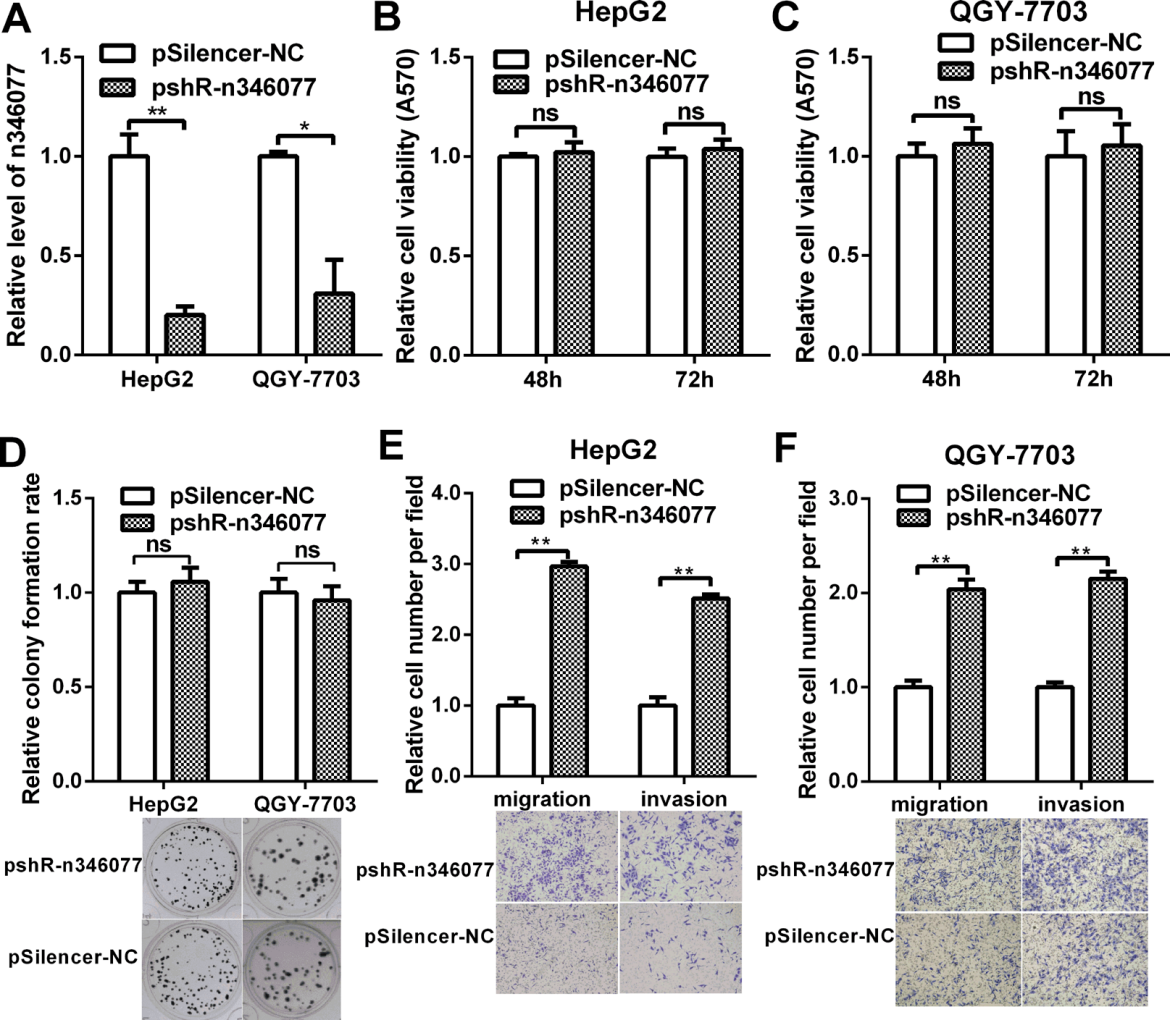
The effect of n346077 knockdown on malignant behavior of HCC cells *in vitro* (**A**) The expression level of n346077 was analyzed by RT-qPCR in HepG2 and QGY-7703 cells after transfection with pshR-n346077. (**B**) and (**C**) MTT assay of both HepG2 and QGY-7703 cells at 48 h, 72 h after transfection with pshR-n346077. (**D**) Colony formation assay was performed in both HepG2 and QGY-7703 cells after transfection with pshR-n346077. Representative images are shown. (**E**) and (**F**) Transwell migration and invasion assays were performed in both HepG2 and QGY-7703 cells after transfection with pshR-n346077. Representative images are shown. *P < 0.05; **P < 0.01.

We then examined the effect of n346077 knockdown on the malignant behavior of these two HCC cells. The knockdown efficiency of the pshR-n346077 was confirmed in HepG2 and QGY-7703 cells (Figure 8A). As shown in Figure 8B–8D, knockdown of n346077 has no effect on the viability and colon formation ability of HepG2 and QGY-7703 cells. But the cell migration and invasion abilities of both HepG2 and QGY-7703 cells were significantly increased when the expression level of n346077 was decreased (Figure 8E and Figure 8F). These results further confirmed its tumor suppressor role in HCC cells.

## DISCUSSION

With the emerging studies focusing on the regulatory role of lncRNAs, it has been found that lncRNA contributes significantly to the pathogenesis and progression of cancers, including HCC [11, 12]. For HBV-related HCC, previous studies are mainly focus on protein-coding genes and miRNAs, the studies on the deregulated expression of lncRNAs and the involvement of lncRNAs just begin. Pan *et al.* and Yu *et al.* analyzed the lncRNA expression profiles between HBV-related HCC tissues and corresponding normal liver tissues using microarrays without characterizing the function of a specific lncRNA, respectively [22, 23]. Gong *et al.* investigated lncRNA expression profiles in HBV-related HCC by annotating and analyzing microarray datasets and identified 182 lncRNAs that were specifically differentially expressed in HBV-related HCC [24]. A recent study reported the expression profile on lncRNA among 5 male HBV-induced HCC cases by using microarray analysis [25]. Yang *et al.* identified the differentially expressed lncRNAs between HBV-related HCC and paired peritumoral tissues by microarray and characterized the oncogenic role of lncRNA-HEIH in tumor progression [15]. However, to our knowledge, systematic characterization of lncRNA in HBV-related HCC is rare. In this study, we generated global lncRNA expression profiles associated with HBV-related HCC using next-generation RNA-seq, which may provide valuable resources to further study the role of lncRNAs in HBV-related HCC development.

As for the first study of genome-wide analysis of lncRNAs expression profiles in HBV-related HCC, we obtained 8757 novel lncRNAs and 14532 known lncRNAs included in NONCODE v3.0 database. The high ratio of novel to known lncRNAs may be due to the rarity of studies of the lncRNAs in HBV-related liver diseases. Further, we identified approximately 654 lncRNAs and 515 mRNAs were differentially expressed in HBV-related HCC. We compared the differentially expressed mRNA and lncRNA with previous studies. For example, BAIAP2-AS1 [24], THBS4 [25] are also over-expressed in our samples, which supports the reliability of our study. Although the difference of BAIAP2-AS1expression did not reach statistical significance, which may be due to the small number of samples analyzed. Moreover, the RT-qPCR results in an independent HBV (+) HCC patients and HepG2.2.15 cells showed the same trends of changes in expression levels with the RNA-Seq results, further confirming the accuracy and reliability of our study. We will confirm our RNA-Seq data in larger cohorts of well-controlled subjects in subsequent research.

In our study, we find that the average expression level of lncRNA transcripts is slightly higher than that of protein-coding genes (Figure 2B). Similar with our findings, a recent study which focus on the effect of HSV-1 on lncRNAs expression showed that the expression levels of lncRNAs are higher than protein-coding genes after HSV-1 infection for 6 and 8 h, which may be due to the degradation of host mRNAs by viral protein UL14 [26–28]. But lncRNAs are generally lower expressed than protein-coding genes by comprehensive analysis of their expression in multiple human organs [8]. The reason for the difference may be due to the expression of some of the HBV proteins, such as HBx, which has been reported to regulate the expression of many protein coding genes and non-coding RNAs [29]. It may also be due to the HBV DNA integration, which leads to 23.1% of protein-coding genes and 24.7% of lncRNAs genes with more than two times change [16, 30]. We will explore the specific mechanism in the future.

Furthermore, we explored the potential function of dysregulated lncRNAs by prediction of their cis- and trans-regulated target genes and subsequently subject them into GO and KEGG analysis. Interestingly, the KEGG pathway analysis showed that the over-represented terms are enriched in many aspects of hepatic metabolic pathways (Figure 6B), which are consistent with the recent reports that focus on the relationship between HBV-related liver diseases and the derangement of hepatic metabolic pathways [31, 32]. However, these predicted functions of lncRNAs require experimental verification. Furthermore, whether the deregulated expression of lncRNAs by HBV may benefit HBV own replication will be investigated in the future in our lab.

A known lncRNA, n346077, encodes a 2609bp transcript and is located in the opposite strand of MRPL23 gene, was one of the most down-regulated lncRNA in HBV-related HCC. To test whether it contribute to the pathogenesis of HCC, we examined its effect on the malignant behavior of hepatomas cells. MTT and colony formation assays showed no significant differences when the expression level of n346077 was changed. Tranwell assays with or without matrigel showed that n346077 overexpression attenuated but n346077 knockdown enhanced the invasion and migration abilities of HepG2 and QGY-7703 cells, revealing that n346077 may act as a tumor suppressor gene in hepatoma cell lines, which explain its decreased expression in HBV-related HCC. It has been reported that MRPL23 can suppress the proliferation of gastric cancer cells and enhance the therapeutic efficacy of adenoviral-mediated p53 gene transfer in models of human gastric cancer by inhibiting MDM2-mediated p53 degradation [33–35]. Since n346077 is located in the opposite strand of MRPL23 gene, whether it functions through augmenting MRPL23 tumor suppressor function will be investigated in the future.

In conclusion, we first profile the lncRNAs expression in HBV-related HCC based on transcriptome RNA-seq approach and provide novel insight into the pathogenesis of HBV-related HCC. Further exploration of the lncRNAs function will be helpful in our understanding the regulatory roles of lncRNAs in HBV-related HCC development.

## MATERIALS AND METHODS

### Patients and tissue samples

Tissues samples from 3 HBV (+) HCC patients and 3 HBV (–) HCC patients were used for deep sequencing. Tissues samples from 10 HBV (+) HCC patients and 3 HBV (–) HCC patients were used for data validation. The liver cancer tissues were snap-freezed in liquid nitrogen immediately after removal and then stored at –80°C before RNA extraction. Written informed consent was obtained from all patients and the study was approved by the Ethics Committee of Tianjin Medical University. Detailed information of the patients is listed in Table 1.

**Table 1:** The clinical and pathological features of the liver cancer patients

ID	sex	age	HBV DNA	Tumor Grade (TNM)
Tumors used for sequencing (*n =* 6)				
HBV (–) HCC				
N1	male	38	-	T1N0M0
N2	male	34	-	T1N0M0
N3	male	34	-	T1N0M0
HBV (+) HCC				
P1	male	30	3.56*10E4	T1N0M0
P2	male	46	1.41*10E4	T1N0M0
P3	male	36	1.05*10E7	T3N0M0
Tumors used for validation (*n =* 13)				
HBV(+) HCC				
V1	male	40	1.04*10E6	T3N0M0
V2	male	42	4.41*10E4	T1N0M0
V3	male	55	1.17*10E6	T4N0M0
V4	female	65	6.12*10E3	T3N0M0
V5	female	51	1.21*10E4	T4N0M0
V6	male	36	2.86*10E3	T2N0M0
V7	male	50	6.37*10E5	T4NOMO
V8	male	53	1.35*10E3	T4NOMO
V9	male	41	4.15*10E4	T1N0M0
V10	male	69	1.63*10E4	T1N0MO
HBV (–) HCC (*n =* 3, the tissues for sequencing)				

### RNA extraction and sequencing

Total RNA was extracted from tissue samples using TRIzol reagent (Invitrogen, MA, USA) according to the manufacturer’s protocol. The preparation of whole transcriptome libraries and deep sequencing were carried out at the Beijing Genomic Institute at Shenzhen (BGI-Shenzhen, Shenzhen, China). RNA-Seq was performed on an Illumina Hiseq 2000 platform and 90 bp paired-end reads were generated according to Illumina’s protocol.

### RNA-Seq data analysis

Raw data were first processed using in-house Perl scripts. In this step, clean data were obtained by removing reads containing adapter, reads containing N for more than 10% of all the bases and low quality reads.

The paired-end clean reads were then aligned to human genome build 19 (hg19) with HISAT, The mapped reads of each sample was assembled using Cufflinks (v2.1.1) in a reference-based approach. For mRNA analyses, the RefSeq database (Build 38) was chosen as the annotation references. For lncRNA analyses, the NONCODE v3 database was chosen as the annotation references.

### Differential expression analysis

The expression level of each transcript was calculated with RSEM and was expressed as FPKM (Fragments Per Kb per Million reads). Volcano Plot, scatter plot filtering and hierarchical clustering were performed to identify differentially expressed lncRNAs and mRNAs with statistical significance. The threshold is Fold Change ≥ 2.0, *P* value ≤ 0.05.

### Prediction of the function of lncRNAs

To explore the functions of lncRNAs, we predict their target genes in cis and in trans. In cis refers to lncRNAs’ action on neighboring target genes. In trans refers to the influence of lncRNAs on other genes at the expression level. In present study, we defined the mRNAs as cis-regulated target genes when (1) it was located within 10 kb upstream or 100 kb downstream in genomic distance from the lncRNA and (2) both Spearman correlation coefficient (SCC) and Pearson correlation coefficient (PCC) ≥ 0.8. We defined the mRNAs as trans-regulated target genes when (1) it is located beyond the above distance in genomic distance from the lncRNA or in different chromosomes.

### GO analysis and KEGG pathway analysis

GO enrichment analyses of differentially expressed genes or lncRNA target genes were implemented by the GO seq R package, in which gene length bias was corrected. KEGG pathway analyses were performed using KOBAS software.

### RT-qPCR

Total RNA was isolated using Trizol (Invitrogen, MA, USA). First strand cDNA was synthesized using Moloney murine leukemia virus reverse transcriptase (Promega, Madison, WI, USA). RT-qPCR analysis was performed with the SYBR Premix Ex TaqTM kit (TaKaRa, Shiga, Japan) according to the manufacturer’s instructions. Samples were normalized using β-actin as an endogenous control. All the primers used are listed in Table 2.

**Table 2:** Primers used in this work

**Primers**	**sequences (5**′ **to 3**′**)**
LTCONS_00067638 forward	GAGCCTGTTGTCCTTTGATTGA
LTCONS_00067638 reverse	TTCTGGGAAATAGTGCTTGATA
LTCONS_00098889 forward	TCACCTGTCAAATCTTCCTCAA
LTCONS_00098889 reverse	TGTCTGCCACATCATTCCTTAT
LTCONS_00082835 forward	TCTCCGTAGGTTCTCCAATG
LTCONS_00082835 reverse	TGGAATGGGATGTGCTGAAT
LTCONS_00055780 forward	ATACATAAAGAACAATGAGGGAGC
LTCONS_00055780 reverse	TTCACGACAAACAGACGAACAC
LTCONS_00076359 forward	TCCACTGGCATTCTATCACCTA
LTCONS_00076359 reverse	GAAGGGATAAGCCTCTGGTCTC
n336878 forward	CTCCATGTAGACTGTGCTCG
n336878 reverse	AGGGCTTGAGTGGATGGGAATA
n380154 forward	GAGAAATGAAAAGGGAGGTC
n380154 reverse	TTGTATCGGGCAAAGGTG
n365126 forward	GGTACTATCAATGGAGGTGGA
n365126 reverse	GATTTCGTGCTTAGTTGCTTTT
n346077 forward	CCCACACACTAGCCCACTGTTC
n346077 reverse	CATGTCCGTGCCAATTCCTCAA
n335657 forward	CGACATGACCACCTTCAGCAAG
n335657 reverse	GTAAGCGTAGCGTTCACCAGAT
n346077-sense	CGCGGTACCGCCCTCACTCCCCAGGCCCA
n346077-antisense	CCGGAATTCCCCCACCAGGGGCTGTGCA
n346077-shR-top	GATCCGACCCTGTGTCTGGAATGTGGCTCGAGCCACATTCCAGACACAGGGTCTTTTTGA
n346077-shR-bot	AGCTTCAAAAAGACCCTGTGTCTGGAATGTGGCTCGAGCCACATTCCAGACACAGGGTCG

### Cell culture and transfection

The human hepatoma cell line HepG2 cells and QGY-7703 cells were cultured in Dulbecco’s modified Eagle’s medium (DMEM) supplemented with 10% fetal bovine serum at 37°C in a humidified atmosphere with 5% CO_2_. Cells were transfected with plasmids using Lipofectamine^TM^ 2000 (Invitrogen, MA, USA) following the manufacturer’s protocol.

### Plasmids construction

The pcDNA3.0 was used to generate n346077 overexpressing plasmid with method as described previously [36]. Briefly, the cDNA of n346077 was amplified from the cDNA of HepG2 cells and then cloned into the KpnI and EcoRI restriction sites of pcDNA3.0 (named n346077). The shRNA oligonucleotides of n346077 was annealed and cloned into pSilencer 2.1-U6 neo vector at BamHI and HindIII sites to construct the knockdown plasmids pshR-n346077. The insertions were confirmed by DNA sequencing. The primers and oligonucleotides used are listed in Table 2.

### Cell viability and colony formation assay

For cell viability assay, HepG2 cells or QGY-7703 cells transfected with n346077 or pshR-n346077 were seeded in 96-well plates and tested using the MTT assay at different time points. For colony formation assay, the transfected HepG2 cells or QGY-7703 cells were seeded in 12-well plates and they were stained with crystal violet and counted when most of the colonies contained at least 50 cells.

### Cell migration and invasion assays

The 24-well Transwell chamber inserts with 8-μm pore size polycarbonate membrane (Corning, Cambridge, MA) without or with Matrigel (for invasion) was used to analyze the migration and invasion of tumor cells. The assays were performed as described previously [36].

## Abbreviations

DMEM: Dulbecco’s modified Eagle’s medium
DREH: down-regulated expression by HBx
GO: gene ontology
HBx-LINE1: HBx-interspersed nuclear element 1
HCC: Hepatocellular carcinoma
HEIH: high expression in HCC
HULC: highly up-regulated in liver cancer
KEGG: Kyoto Encyclopedia of Genes and Genomes
lncRNAs: long non-coding RNAs
MRPL23: mitochondrial ribosomal protein L23
MVIH: microvascular invasion in HCC
RNA-Seq: RNA sequencing
RT-qPCR: quantitative real-time PCR
